# Using sensitivity analyses to understand bistable system behavior

**DOI:** 10.1186/s12859-023-05206-2

**Published:** 2023-04-06

**Authors:** Vandana Sreedharan, Upinder S. Bhalla, Naren Ramakrishnan

**Affiliations:** 1 Genetics, Bioinformatics, and Computational Biology, Virginia Tech, Blacksburg, VA 24061 USA; 2 National Centre for Biological Sciences, TIFR, Bellary Road, Bangalore, 560065 India; 3 Department of Computer Science, Virginia Tech, Arlington, VA 22203 USA

**Keywords:** Eigenvalue sensitivity, Sensitivity analysis, Parameter design, Steady state separation, Bistable switching, Distance to bifurcation

## Abstract

**Background:**

Bistable systems, i.e., systems that exhibit two stable steady states, are of particular interest in biology. They can implement binary cellular decision making, e.g., in pathways for cellular differentiation and cell cycle regulation. The onset of cancer, prion diseases, and neurodegenerative diseases are known to be associated with malfunctioning bistable systems. Exploring and characterizing parameter spaces in bistable systems, so that they retain or lose bistability, is part of a lot of therapeutic research such as cancer pharmacology.

**Results:**

We use eigenvalue sensitivity analysis and stable state separation sensitivity analysis to understand bistable system behaviors, and to characterize the most sensitive parameters of a bistable system. While eigenvalue sensitivity analysis is an established technique in engineering disciplines, it has not been frequently used to study biological systems. We demonstrate the utility of these approaches on a published bistable system. We also illustrate scalability and generalizability of these methods to larger bistable systems.

**Conclusions:**

Eigenvalue sensitivity analysis and separation sensitivity analysis prove to be promising tools to define parameter design rules to make switching decisions between either stable steady state of a bistable system and a corresponding monostable state after bifurcation. These rules were applied to the smallest two-component bistable system and results were validated analytically. We showed that with multiple parameter settings of the same bistable system, we can design switching to a desirable state to retain or lose bistability when the most sensitive parameter is varied according to our parameter perturbation recommendations. We propose eigenvalue and stable state separation sensitivity analyses as a framework to evaluate large and complex bistable systems.

**Supplementary Information:**

The online version contains supplementary material available at 10.1186/s12859-023-05206-2.

## Background

Many important biological mechanisms have an underlying molecular interaction network which enables them to reside in potentially two stable states. Such bistable networks orchestrate the two differentiated states of a cell [1, 2] or two states of a biochemical pathway [3, 4], depending on various external or internal factors. Cellular processes governed by bistable networks include cellular differentiation, the MAPK signal transduction cascade, cell cycle regulation [5–7] and programmed cell death or apoptosis [8, 9]. Studies on such fundamental mechanisms subject to heavy regulation by bistable systems lead to a hypothesis that any damage to the bistable machinery can potentially lead to undesirable outcomes for the cell. Recent advances in cancer research show that the apoptotic bistable system is mutated in malignant cells [10, 11], and there is considerable optimism in studies related to inducing cell death to control tumor progression and to improve treatment response [12, 13]. Similarly, Alzheimer’s disease which is a progressive neurodegenerative disorder has an underlying bistable system which has switched from a normal, healthy state to an irreversible pathological state, with the threshold-like transition after a slow accumulation of symptoms [14, 15]. Such observations warrant fine-grained control over the bistable systems to design the parameters in such a way that the cell reaches desirable outcomes. Such accomplishments can help in developing therapeutic protocols, for instance, to improve efficacy of anticancer therapies in re-activating bistability, and hence apoptosis, in an otherwise apoptosis-resistant cell [16–18].

Many such biological bistable systems have been mathematically modeled and our observation is that only a fraction of those mathematical models are investigated experimentally, such as [7, 19]. In-depth analysis of mathematical models reveals insights for biologists to design their experiments [20]. The tools and techniques available to systems biologists to analyze bistable systems are commonly centered around bifurcation analysis and time course simulations. Observations drawn using bifurcation analysis are either qualitative or visual and they predominantly draw inferences about system properties [21]. Analyzing a large collection of bistable systems and their parameters becomes practically strenuous and this requirement demands metrics and pipelines that are both scalable and quantitative. In this work we provide new analysis methods for bistable systems to determine measures that can provide quantitative insights and help identify parameters to push the system to a desirable cellular decision, as in the case of triggering apoptosis in a tumor cell.

The term sensitivity has different interpretations depending on context and sub-discipline. One interpretation concerns with finding an input to the system at the lowest resolution that can create a significant change in an output; this is practiced in studies involving assays [22]. Another approach to sensitivity analysis is to determine the input–output relationship of a system by varying input signals or parameters as proposed by Goldbeter and Koshland in their classic work [23, 24]. Such an analysis has various applications: as an example, a dynamic sensitivity analysis on NF-$\kappa$B pathway revealed that only a subset of parameters has significant influence on the system oscillations [25]. Similarly, Von Dassow et al. [26] investigated the possibility of whether insect segmentation is a modular process and whether each module has an intrinsic response to an external transient stimulus. They systematically analyzed the system’s sensitivity to initial conditions and observed that the topology of the segmentation network can create multiple solutions that are robust to input fluctuations. Thus, in many applications, sensitivity analysis can play a role in model validation, model reduction, parameter estimation, and experimental designs [25]. Comprehensive summaries of various sensitivity analysis techniques are discussed in [27–30].

In this work we propose to do eigenvalue sensitivity analysis as well as steady state separation sensitivity analysis. Eigenvalue sensitivity analysis is an established technique in engineering. First- and second-order eigenvalue sensitivity have been used to identify stability problems and weak lines in power systems [31]. Information about sensitivity of dominant eigenvalues is frequently used in mechanical structural analysis for dynamic improvements [32, 33]. Root-locus analysis is a classical technique, based on the change of eigenvalues of a system w.r.t. parameters or gains, and is used to tune feedback control systems [34]. While these approaches are commonplace within the engineering community, they are not too frequently applied in systems biology or to gain insights for tuning a bistable system or for parameter design. One such application is eigenvalue analysis on a cell cycle model [21] where the authors divided the temporal evolution of the system into different phases based on the sign of the underlying Jacobian’s eigenvalues. Also, there have been classical approaches in engineering disciplines to combine sensitivity of eigenvalues and eigenvectors w.r.t. system parameters to optimize designs for specified design criteria [35, 36]. These have been studied in engineering using approaches that differ based on the independent variables (state or design parameter), the sensitivity computation method, the set of eigenvectors considered (right or left), and the application domain; however, application of these methods in biochemical reaction networks is limited. For instance, [37] computes the eigenvalue sensitivities of network edges based on random stochastic disturbances in the Jacobian matrix and stops short of providing insight into the critical parameters that are key to stabilizing or destabilizing the network.

Eigenvalue analysis can be used to detect bistability using constrained optimization with the determinant of the Jacobian as the objective function as in [38]. For the sensitivity analyses proposed in this work, existence of bistability is necessary without having to consider how they are detected. While classical input–output sensitivity analysis for a model could potentially measure the steady state concentration change w.r.t. change in parameter, such analysis is not able to provide information regarding the parameter that needs to be modified to influence the stability characteristics of the system. Such insight regarding stability based on parameter sensitivity is a contribution of our analysis.

In this work we implement two measures of sensitivity to mathematical models representing bistable systems. We use the sensitivity analysis principle proposed by Ferrel [39] to compute input and output sensitivity using fold changes. While this formulation has been used for input/output sensitivity, to the best of our knowledge this formulation has not been used frequently for eigenvalue sensitivity analysis on bistable systems before. To evaluate the sensitivity of each parameter, we perturb the parameter, and compute two sensitivity values: eigenvalue sensitivity and stable state separation sensitivity, and list out reactions associated with most sensitive parameters. Biologically, the sensitive reactions are the points of regulation. Sensitive reactions suggest the identification of processes frequently mutated in disease or potentially modifiable using therapeutic drugs [40]. We demonstrate the proposed quantitative analysis technique using the smallest published bistable system [41] as well as a larger bistable system [42]. We selected the smallest system because its lowest dimensionality as a bistable system helps in demonstration and validation of our approach. We also illustrate that the analyses are scalable and generalizable and propose this approach as a framework to analyze more complex databases of bistable systems [42]. Furthermore, our sensitivity analysis pipeline can potentially enhance experimental validation of bistable mathematical models through effective parameter design.

## Results

We introduce two sensitivity measures in this work, namely eigenvalue sensitivity and stable state separation sensitivity. We define eigenvalue sensitivity as the measure of the rate of change of stability of a bistable system w.r.t. parameter changes. We use stable state separation sensitivity as a measure of “goodness” of a bistable switch. Together, the two measures can assist in efficiently designing the system parameters to achieve desired state transitions.

**Fig. 1:**
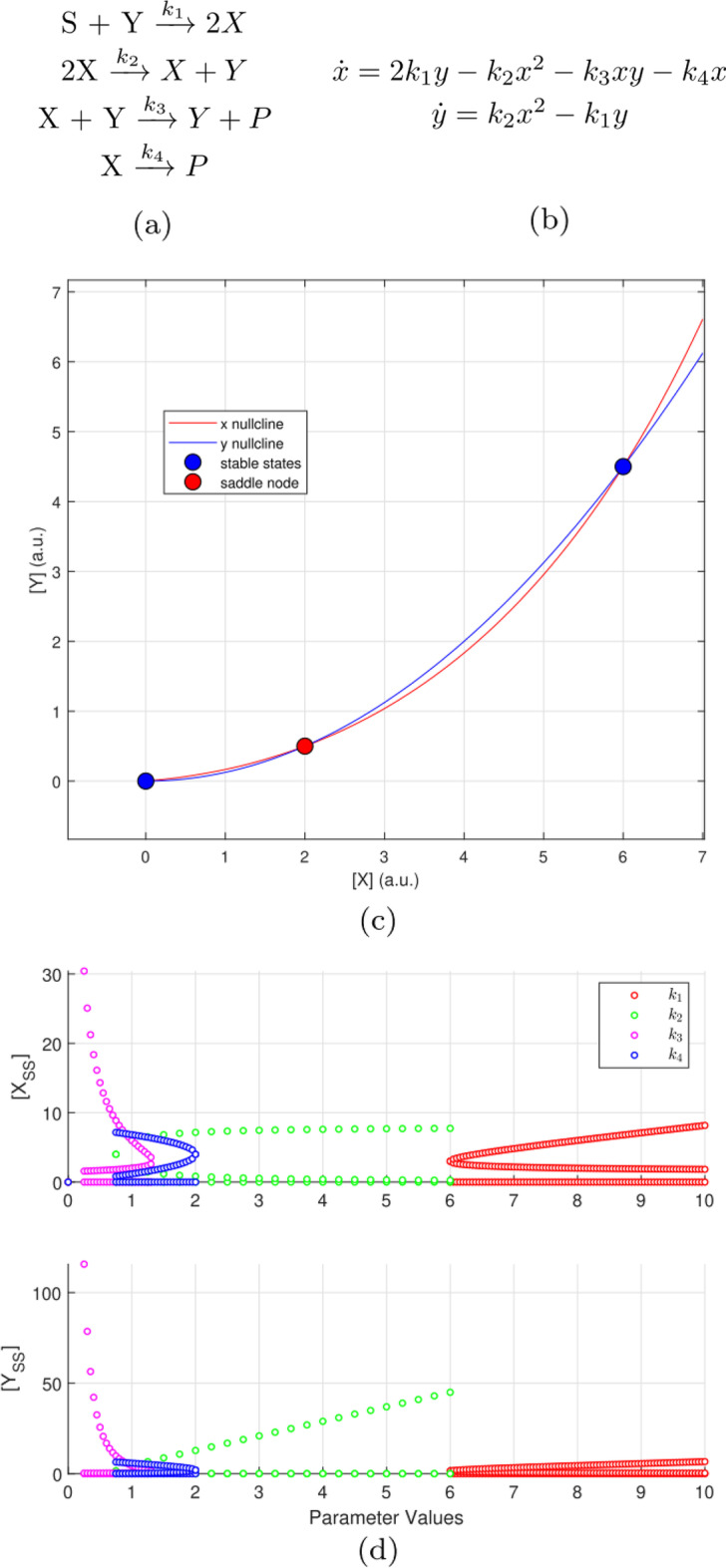
The smallest bistable chemical system proposed by Wilhelm [41]. In this work, we applied eigenvalue and steady state separation sensitivity analyses on this system. (a) Chemical reactions where species concentrations [*X*] and [*Y*] are considered to be the two states of the system. (b) Reaction rate equations where *x* and *y* are the time-dependent states of the system. The reaction rate constants $k_1$, $k_2$, $k_3$, and $k_4$ are non-dimensional. (c) x- and y-nullclines of the system, showing the two stable steady states (blue markers) and saddle node (red marker) at the points of intersection. (d) One-parameter bifurcation diagrams (or S-curves) corresponding to parameters $k_1$ through $k_4$. The width of the S-curve for each parameter represents the bistable region. (See Additional file 1 for more details of the system)

**Table 1:** Summary of eigenvalue analysis on the simplest bistable system [41]

	Solution 1	Solution 2	Solution 3
Steady States (*x*, *y*)	(0,0)	(2, 0.5)	(6, 4.5)
Jacobian $\textbf{J}$	$$\left( \begin{matrix} -1.5 &{} 16\\ 0 &{} -8 \end{matrix}\right) $$	$$\left( \begin{matrix} -6 &{} 14\\ 4 &{} -8 \end{matrix}\right) $$	$$\left( \begin{matrix} -18 &{} 10\\ 12 &{} -8 \end{matrix}\right) $$
Eigenvalues $(\lambda _1, \lambda _2)$	$(-1.5,-8)$	$(-14.5,0.54)$	$(-25.04,-0.95)$
Stability Property	Stable	Saddle node	Stable

For each steady state solution, the corresponding Jacobian, eigenvalues, and stability are listed. There are two stable steady states, which make this system bistable

We applied these sensitivity analyses to a published two-molecule bistable system [41]. A detailed description and dynamical systems analysis of that system, referred to as the smallest bistable system, is provided in the Additional file 1. The chemical reactions, reaction rate equations, null-clines and the bifurcation diagrams for this system are shown in Fig. 1 and summary of eigenvalue analysis is given in Table 1.

### Measurement of change in stability and goodness of a bistable switch

We quantified the change in stability of the system in Fig. 1 as parameters are varied from their nominal values, by computing the sensitivity w.r.t. parameter $p_j$ of the spectral abscissa $\alpha _i$ for each stable steady state *i* in the bistable system ($i \in \{1,2\}$) using centered difference estimate:

1 $$\begin{aligned} {\hat{m}}_{ij} = \left( \frac{\alpha ^{+}_{i} - \alpha ^{-}_{i}}{2 \epsilon _j p^{*}_j} \right) \frac{p^{*}_j}{\alpha ^{*}_i} \end{aligned}$$

Here $\alpha ^{\{\}}_{i} = \max \left[ \mathfrak {Re} \left( \pmb {\lambda }_i \right) \right] ^{\{\}}$ are the spectral abscissa [43], i.e. largest real part of all eigenvalues $\pmb {\lambda }_i$, for each stable steady state *i* for percentage perturbations $\epsilon _j$ to either side, positive and negative, of the nominal parameter values $p^{*}_j$. The mathematical operator $\mathfrak {Re}(z)$ denotes the real part of the complex number *z*. We chose the largest real part, $\alpha ^{+}_i$ and $\alpha ^{-}_i$, for parameter perturbations to either side of the nominal value because it principally governs the stability characteristics of a given stable steady state. For simplicity, we use the term “eigenvalue sensitivity” to mean spectral abscissa sensitivity and “maximum eigenvalue” to mean the spectral abscissa.

**Fig. 2:**
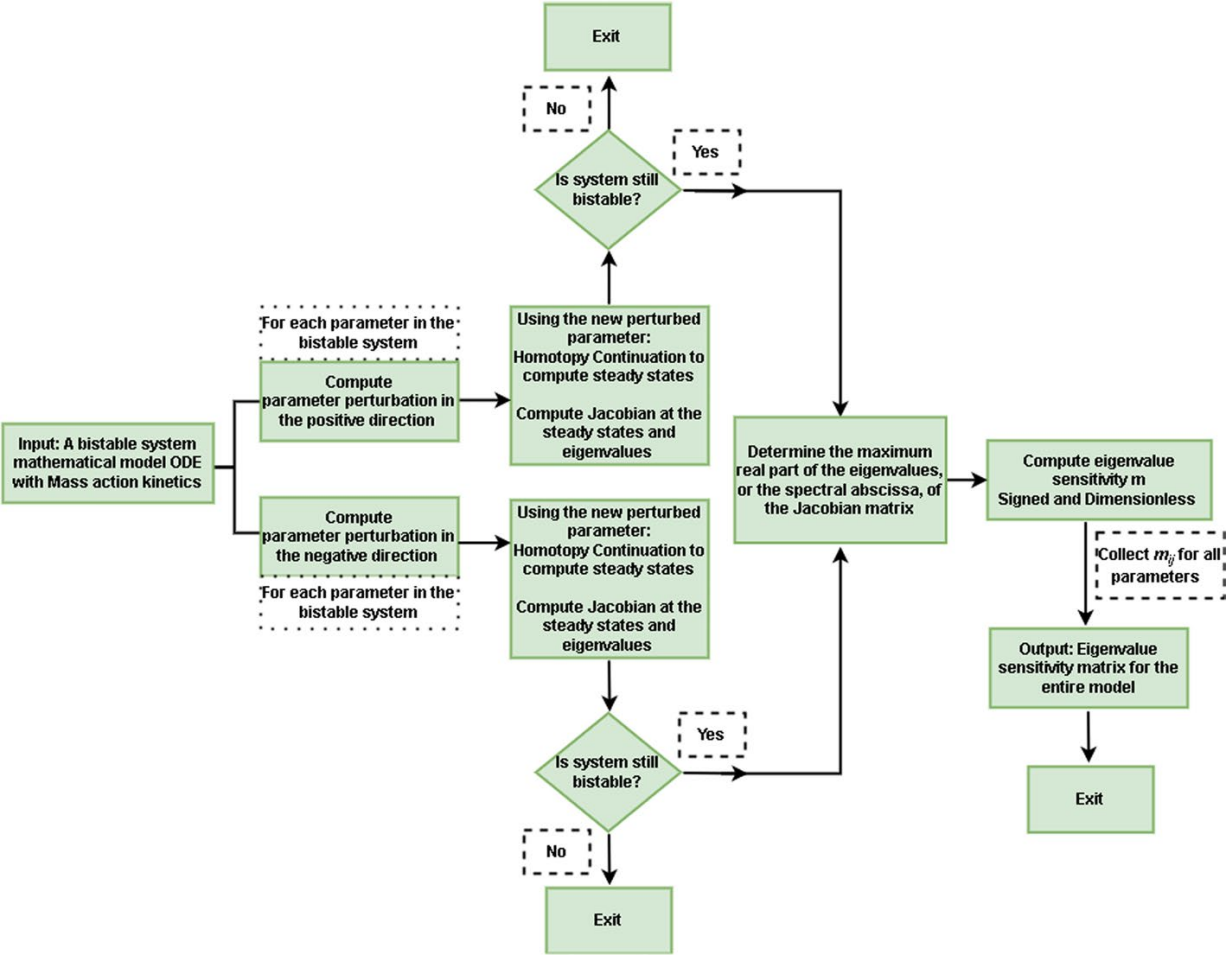
Eigenvalue sensitivity analysis flowchart. A mass-action kinetics model of a bistable system is perturbed in both positive and negative directions of a nominal parameter setting. Using the centered-difference method, the rate of change in eigenvalue w.r.t change in parameter is computed. The above workflow is repeated for all parameters one at a time. Based on the eigenvalue sensitivity, parameters can be clustered automatically. The clusters corresponding to the higher eigenvalue sensitivities are prime candidates to modify the stability characteristics of the system via parameter design

The eigenvalue sensitivity measure ${\hat{m}}_{ij}$ is dimensionless, signed, and represents a polynomial exponent of the functional relationship between the maximum eigenvalue and parameters. *The sign of* ${\hat{m}}_{ij}$ *suggests whether the parameter stabilizes (positive) or destabilizes (negative) the stable state when this parameter is increased and its magnitude suggests the influence this parameter has over the maximum eigenvalue*. The procedure is summarized in the flowchart in Fig. 2. See the methods section for more details.

Characteristics of a good switch have been discussed previously [44]. It is well understood that a bistable switch becomes more immune to noise or fluctuations when the two stable states are maximally separated. We used the Euclidean distance $\Delta SS$ to measure the separation between two stable steady states, $SS_1$ and $SS_2$. Sensitivity of stable state separation $\Delta SS$ with respect to variation in parameters $k_j$ was computed as:

2 $$\begin{aligned} {\hat{s}}_j= \frac{(\Delta SS^{+} - \Delta SS^{-})}{2 \epsilon p^{*}_j} \frac{p^{*}_j}{\Delta SS^{*}} \end{aligned}$$

Stable state separation sensitivity ${\hat{s}}_j$ (called separation sensitivity hereafter for brevity) is signed and dimensionless, just like eigenvalue sensitivity. However, separation sensitivity is different from eigenvalue sensitivity in that the former is a property of the entire network while the latter is applicable only to a given stable state.

### Quantitative analysis of parameter influence on stability and goodness of a switch

**Fig. 3:**
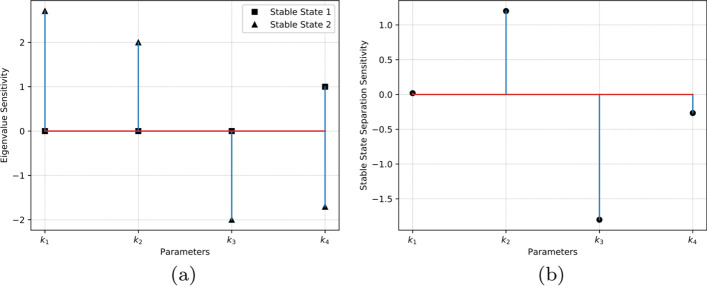
Summary of sensitivity analyses for the smallest bistable system (a) Eigenvalue sensitivity ${\hat{m}}$ for stable state 1 and 2 ($SS_1$, $SS_2$), with parameter perturbations for $k_1$ through $k_4$. For $SS_1$ (0, 0) the maximum eigenvalue is influenced only by $k_4$; $k_1$, $k_2$, and $k_3$ do not influence it (shown by the zero eigenvalue sensitivity). For $SS_2$ (6, 4.5), $k_1$ and $k_2$ perturbations in the positive direction stabilize it while a similar change in $k_3$ and $k_4$ destabilizes it. (b) Sensitivity of separation between stable steady states for the system. Parameter $k_1$ has minimal effect on the goodness of this switch. When perturbed in the positive direction, parameter $k_2$ increases the separation between $SS_1$ and $SS_2$. Parameters $k_3$ and $k_4$ both need to be perturbed in the negative direction to increase separation, $k_3$ having the largest influence. For a simple system such as the one investigated here, these trends are visually evident in the one-parameter bifurcation curves in Fig. 1d

The results for the application of eigenvalue sensitivity analysis to the smallest bistable system are shown in Fig. 3. This figure shows the eigenvalue sensitivity of both stable states for all parameters at the nominal parameter setting of the model.

The eigenvalue sensitivity for $SS_1$ does not change except w.r.t $k_4$. This finding from our analysis is insightful to design parameters in the system and can be validated as follows for a simple system such as this. The Jacobian $\textbf{J}$ for $SS_1$ is already in echelon form (see Additional file 1) which suggests that the diagonal elements $\textbf{J}_{11}$ and $\textbf{J}_{22}$ are the eigenvalues. Among these eigenvalues, $\textbf{J}_{11}$ is maximum because $k_4<k_1$ (refer the nominal parameter values in the Additional file 1). So, the maximum eigenvalue is only affected by $k_4$ and linearly so, as correctly measured by the eigenvalue sensitivity shown by Fig. 3a (if the eigenvalue sensitivity is unity, then the maximum eigenvalue is a first degree polynomial function of the given parameter). However, this linear dependence is not evident from inspecting the bifurcation plot (or s-curve) shown in Fig. 1d. This observation suggests that eigenvalue sensitivity can potentially expose stability behavior that may not be observable from the s-curve. Also, for $SS_1$, the analysis points to $k_4$ as the only means to “change” the system to make a transition to monostable region because the other parameters do not govern the dominant eigenvalue of the system.

The results of separation sensitivity are shown in Fig. 3b. The insights from our quantitative analysis discussed below can be verified visually from the one-parameter bifurcation plots (Fig. 1d) for a simple system such as the one investigated here:

- $k_1$ does not significantly control the separation of stable states of this switch. Among all the s-curves, the vertical width of the red curve corresponding to $k_1$ is the least.
- The separation between $SS_1$ and $SS_2$ increases when changes in $k_2$ are positive. When the separation between stable states increases, the switch becomes more immune to noise and hence the goodness of the switch improves. The green s-curve (for $k_2$) has a net positive slope indicating increasing stable state separation as $k_2$ is increased.
- The separation sensitivity magnitude is the largest for $k_3$ which indicates that it is the parameter that most controls the noise immunity of the network; however we know from Fig. 3 that $k_3$ needs to be decreased to make the system more robust and stable. The magenta s-curve for $k_3$ is a laterally inverted “S” and has a large negative slope.
- Parameter $k_4$ behaves similar to $k_3$ except that its influence on robustness is relatively less. The blue s-curve for $k_4$ is also a laterally inverted “S” with negative slope.

### Automatic clustering of parameters based on sensitivities

**Fig. 4:**
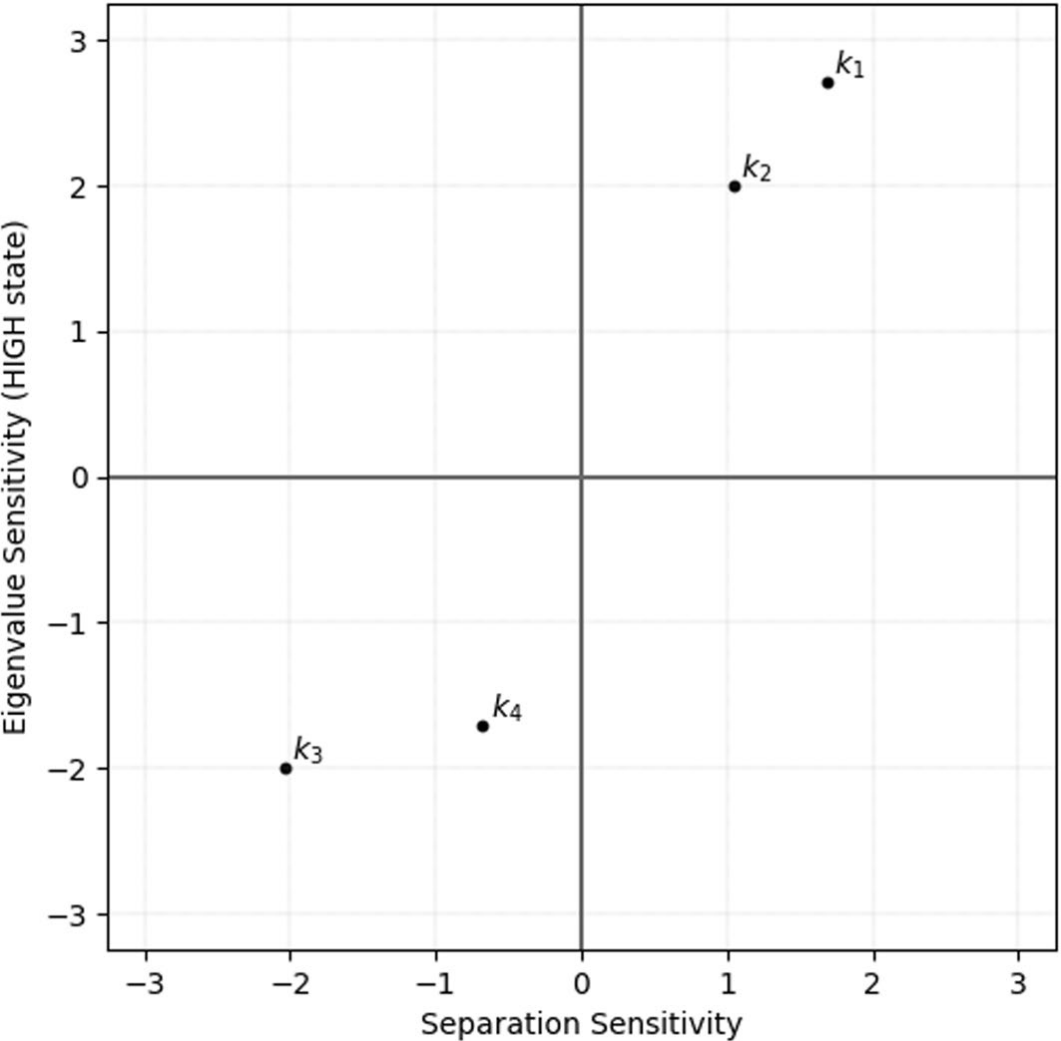
Eigenvalue and separation sensitivity plotted in a sensitivity space. There is positive correlation between the two sensitivities. The clusters $\{k_1,k_2\}$ and $\{k_3,k_4\}$ correspond respectively to S-curve and laterally inverted S-curve

The sign of eigenvalue and separation sensitivity measures naturally clusters the parameters into meaningful sets. For instance, when these measures for the minimal bistable system are plotted in a sensitivity space as shown in Fig. 4, there is a positive correlation between the two sensitivities and the parameters are clustered into two sets in the first and the third quadrants.

Specifically, eigenvalue sensitivity analysis on all parameters for a stable state automatically yields clusters of parameters that stabilize and de-stabilize the given stable state. The cluster $\{k_1, k_2\}$ (and therefore, reactions 1 and 2) of parameters with positive eigenvalue sensitivity (${\hat{m}}_{ij}$) tend to stabilize $SS_2$ when increased. This means that when $\{k_1, k_2\}$ are perturbed in the positive direction at *SS*_2_, this stable state of the bistable switch is further stabilized; however when they are perturbed in the negative direction this stable state is destabilized, ultimately resulting in a monostable system after the bifurcation point. The above behavior is the opposite for the cluster $\{k_3, k_4\}$ which has negative values of ${\hat{m}}_{ij}$. A similar argument about parameter perturbation directions can be made based on the sign of separation sensitivity, except that this measure applies to the bistable switch rather than to a given stable state.

**Table 2:** Summary of parameter perturbation rules

**Condition**	**Stabilize**	** De-stabilize**	**Parameter cluster for** $SS_2$
${\hat{m}}_{ij} < 0$	Decrease *k* $\downarrow$	Increase *k* $\uparrow$	$\{k_3,k_4\}$
${\hat{m}}_{ij} > 0$	Increase *k* $\uparrow$	Decrease *k* $\downarrow$	$\{k_1,k_2\}$

**Condition**	**Increase**	**Decrease**	**Parameter cluster**
	**separation**	**separation**	**for switch**
${\hat{s}}_{j} < 0$	Decrease *k* $\downarrow$	Increase *k* $\uparrow$	$\{k_3,k_4\}$
${\hat{s}}_{j} > 0$	Increase *k* $\uparrow$	Decrease *k* $\downarrow$	$\{k_1,k_2\}$

Enumeration of parameter perturbation directions to: (i) stabilize or de-stabilize a given stable state (based on the sign of ${\hat{m}}_{ij}$) and (ii) increase or decrease the separation between stable states (based on the sign of ${\hat{s}}_{j}$). Parameter clusters corresponding to the stated conditions are also listed. Note that the trends for clusters based on both sensitivities are similar. The symbol $\uparrow$ indicates an increase in the stability property listed for that column and $\downarrow$ indicates the decrease thereof

The above information is summarized in Table 2. The trends for parameter clusters based on both sensitivities are similar because in effect these are clusters of “S-” and laterally-inverted “S” curves shown in Fig. 1d. The eigenvalue sensitivity measure table is based on the interpretation described later in Fig. 11 (see Methods section).

When a system with an intractably large number of parameters needs to be changed to make desirable state transitions, it can be hard to visually inspect the parameter bifurcation diagrams and make qualitative decisions. In such cases, parameter clustering based on quantitative sensitivity analyses can be valuable, as discussed in the next subsection.

### Dominant parameter cluster can be used to switch efficiently

By considering both eigenvalue sensitivity and separation sensitivity, we can identify the parameters that should be modified to achieve a particular switching goal. The different parameter design rules were summarized in Table 2. The rules can be used for modifying the stability of a given stable state and separation of the two stable states for desirable outcomes. The relevance of parameter design for systems biology applications has been discussed by Murphy et al. [45].

To demonstrate the benefits of the parameter design rules, we considered the scenario of switching from $SS_2$ in the bistable system to a monostable state after bifurcation. The motivation was to answer the following questions:

- What is the lowest parameter perturbation that can trigger the system state to switch from bistable to monostable system?
- Which parameter cluster does this lowest perturbation correspond to?
- Is there a correlation between most sensitive parameter cluster and the cluster that causes switching?
- How does the behavior of the deterministic model compare with that of a population of models, with regard to switching?

#### Validation of sensitivity analyses using a distance to “cliff” measure

A bistable system switches from the nominal ON state (in the bistable region) to a monostable state when the nominal parameter setting is progressively perturbed to the bifurcation point and eventually collapses into a monostable region. In order to validate our sensitivity analyses results - to evaluate which parameter pushes the system away from bistable region with the minimum perturbation - we defined a measure, *distance to the cliff*. This measures the distance of a perturbed model to the bifurcation point along the bifurcation curve. This measure was then used to determine the parameter(s) that will trigger a transition to the monostable state with the lowest perturbation.

**Fig. 5:**
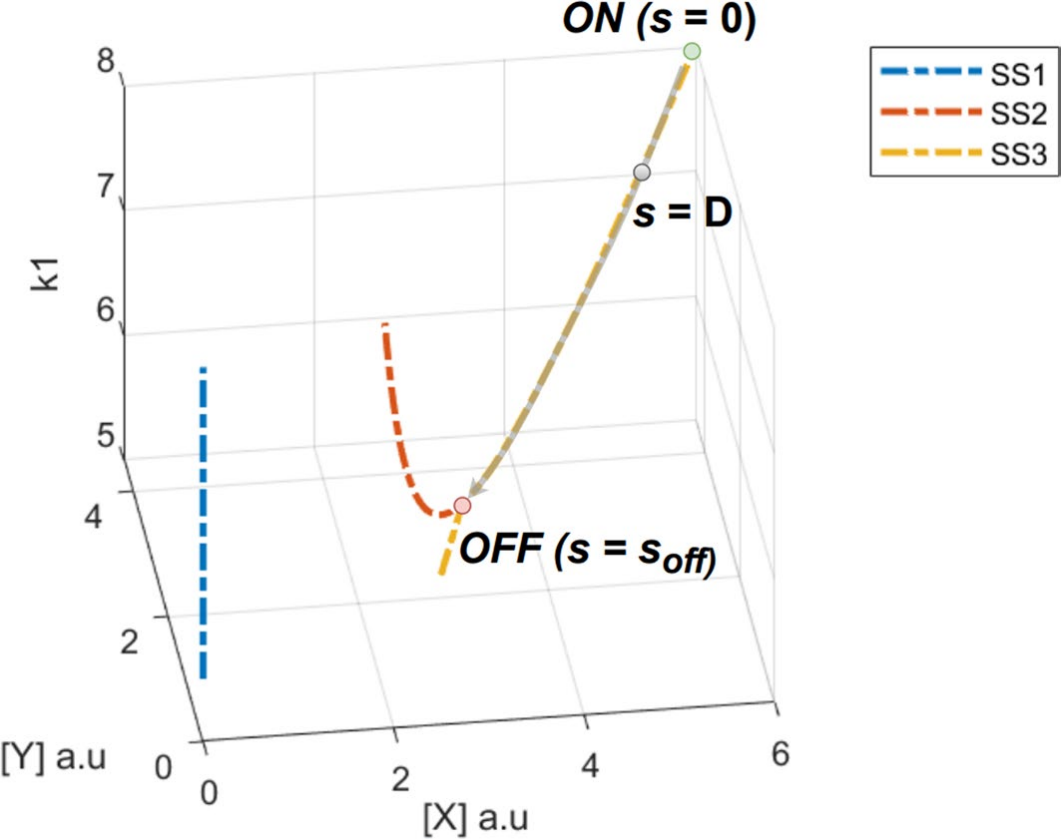
Arc length measure in parameter-state space. The parameter-state space consists of the perturbed parameter (one at a time) and the concentrations of the participating species. The bifurcation curve shown is in this space. Initial ON state corresponds to the nominal system model ($s=0$). After a non-zero perturbation along the $k-$axis, the system moves by an arc length $s=D$. When further perturbed, the system is eventually pushed to the bifurcation point ($s=s_{off}$) where it transitions to a monostable system at the bifurcation point labeled OFF

To compare the system responses for various parameter perturbations, in our simulations we used the arc length (*s*) in parameter-state space to define the distance to the cliff. With reference to Fig. 5, consider a model beginning in the ON state (where $s=0$) which undergoes a perturbation in a given parameter, say $k_1$, that results in a new steady state (where $s=D$). The arc length *s* is measured along the curve in parameter-state space that the system follows as the parameter $k_1$ is perturbed until it arrives at the bifurcation point, labeled OFF in Fig. 5, where $s = s_{off}$.

The system switches to a monostable state at the bifurcation point and hence results in a switching of the stable state to the monostable region. In other words, the maximum value of *s*, $s_{max}$, before the system transitions to monostable region can be interpreted as a distance from the nominal model to the cliff (or bifurcation point). If the distance $s_{max}$ is known for a particular parameter, then the percent arc length is computed as $s_r=s/s_{max}$. In this case, the normalized distance to the cliff is computed as $s_c = 1-s_r$. (See the Methods section for details.)

**Fig. 6:**
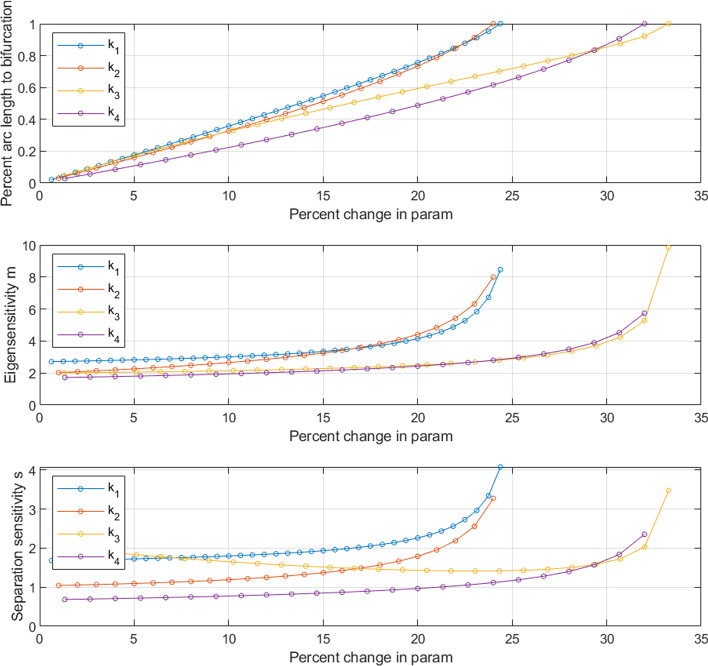
Sensitivity analyses agree with arc length measure in predicting switching. Relationship between eigenvalue sensitivity, separation sensitivity, and percent arc length to parameter perturbation. (Top) Arc length ratio is a definitive measure of when switching occurs ($s/s_{max} = 1$). This condition is first attained for perturbations $\approx 25\%$ for $\{k_1, k_2\}$. For $\{k_3, k_4\}$ this transition occurs after further perturbation of $\approx 30-35\%$. Therefore, $\{k_1, k_2\}$ is dominant parameter cluster in that lesser amount of perturbations in these parameters can lead to switching. (Middle) The vertical ordering of eigenvalue sensitivity curves indicates that the clustering observed in the arc length plot is reproduced.(Below) The trend seen with eigenvalue sensitivity is repeated with separation sensitivity results as well. This shows that the sensitivity analyses can be used as a proxy to predict which parameters will cause switching first when perturbed by the same amounts

For deterministic simulations of a single cell modeled as the smallest bistable system, we tracked eigenvalue sensitivity, separation sensitivity, and percent arc length as the parameters are all perturbed by similar percent changes (Fig. 6).

In all three plots in Fig. 6, the same parameters reached the monostable region with lower perturbation. Parameters $k_1$, $k_2$ switch with lower percent change while $k_3$, $k_4$ switch much later with relatively higher perturbations. Consistency of this clustering pattern shows that eigenvalue sensitivity and separation sensitivity measures are able to predict which parameters will cause the transition to the monostable region. In other words, they indicate quantitatively “how bistable” the system is. This verifies that eigenvalue and separation sensitivity analyses can be used as proxies to determine the dominant parameter cluster that dictate switching.

#### Dominant parameter cluster for a population and a single model correlate

**Fig. 7:**
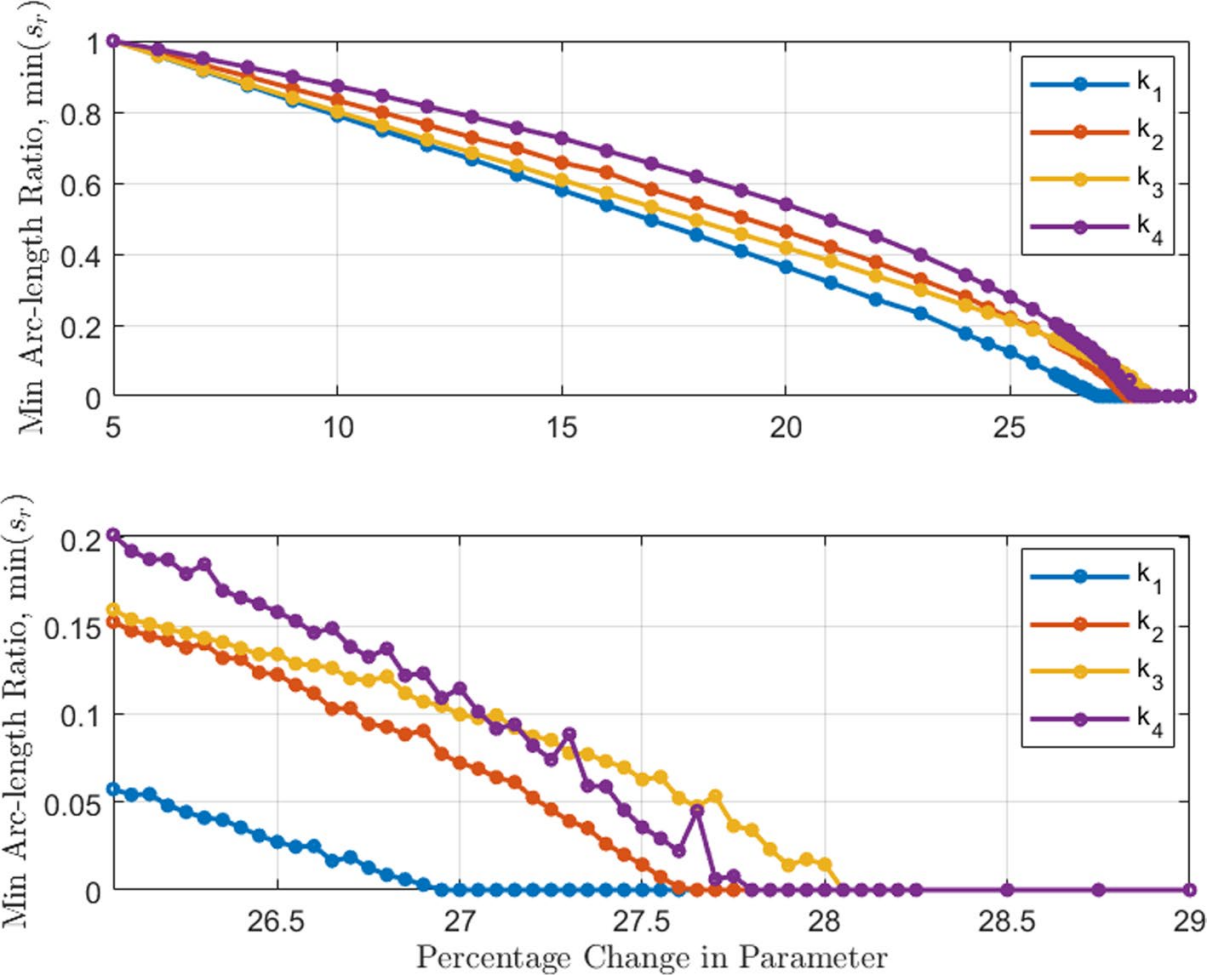
Arc length and parameter perturbation at the population level. Maximum arc length ratio vs. percentage change in parameter value for a population of 20, 000 models. Each setting for a given parameter draws from a log-normal distribution with $3\sigma$ equal to the percent change. This generates an output distribution of arc length ratios, the maximum of which is considered. The parameter cluster $\{k_1,k_2\}$ causes transition to the monostable state the earliest when compared to $\{k_3, k_4\}$. Lower panel shows a zoomed-in view of the upper panel capturing the instances where perturbation in each parameter achieves bifurcation

We validated that the dominant parameter cluster at the population level correlates to that from a single model presented above. We used the Monte Carlo method to determine the output distribution of percent arc length (see Methods section for details) for an input distribution of parameter values sampled from a log-normal distribution. This was performed for a population of 20, 000 models. Such parameterization has been used in the literature [42, 46] and ensures positive values for reaction rates. The mean and three standard deviations ($3\sigma$) of the input distribution were chosen to be the nominal parameter value and the percentage perturbation, respectively. Such systemic noise in reaction rate constants captures cell-to-cell variations [47]. The arc length percent measures were computed using deterministic simulation of the system for each sampled parameter setting. The maximum value of the output distribution (arc length percent distribution) that resulted from the perturbation for a given parameter was used to represent the proximity to the bifurcation point at that setting. This technique was repeated for multiple amounts of perturbation and the results are shown in Fig. 7. The parameter cluster $\{k_1,k_2\}$ first cause the population to switch to the bifurcation point at a perturbation value $\approx 27\%$, indicating that this is the dominant parameter cluster. At the population level, the parameter set $\{k_3,k_4\}$ also transition the system to the bifurcation point soon after but at a larger perturbation value $\approx 28\%$. This shows promise that the eigenvalue and separation sensitivity analyses can potentially predict the dominant parameter cluster for a population of cells. (Eigenvalue and separation sensitivity analyses at the population level are not included in this work).

### Local sensitivity analysis is consistent with global trend within bistable region

**Fig. 8:**
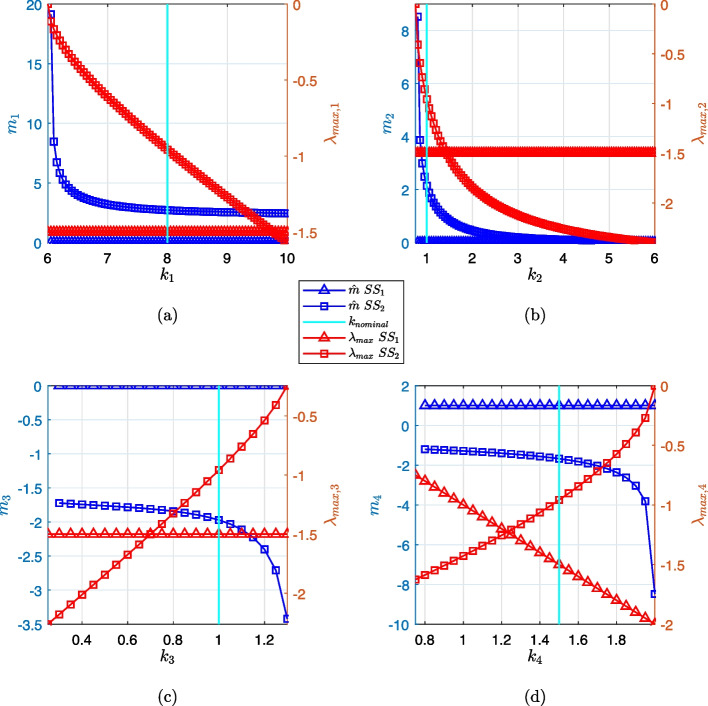
Comparison of local eigenvalue sensitivity analysis and its global trend within the bistable region. The smallest bistable system’s maximum eigenvalue (red markers) and eigenvalue sensitivity (blue markers) are plotted as a function of each system parameter (reaction rate constants $k_1$ through $k_4$) taken one at a time while others are retained at their nominal values. The parameter values span the system’s bistable region. The vertical cyan line shows the nominal parameter setting ($k_{nominal}$) and the local eigenvalue sensitivity analysis at this setting was shown earlier in Fig. 3a. The design rules presented earlier in Table 2, which was based on the local sensitivity analysis, are verified here to correlate with the global sensitivity trend: (a) and (b) show $\{k_1, k_2\}$ stabilize the system (slope of the eigenvalue sensitivity curve decreased) as the parameter value is increased from the nominal setting. (c) and (d): $\{k_3, k_4\}$ are de-stabilizing as the parameter value is increased from the nominal setting

The analysis thus far has only utilized a numerical estimate of eigenvalue sensitivity at the nominal parameter setting. For the smallest bistable system, we computed the eigenvalue sensitivity measure for the range of parameter settings spanning the width of the bifurcation curves that were shown in Fig. 1d, representing the global behavior of this measure. For a two-component system, this is computationally tractable, but a larger system may need significant computational power to accomplish the same task (because each parameter setting requires the solution of three eigenvalue problems). In Fig. 8, we have shown results of the local vs. global behavior of eigenvalue sensitivity.

For this system, the maximum eigenvalue ($\lambda _{max}$) at any given steady state was a continuous function of the parameter, the curves with red markers in Fig. 8, which made it possible to compute a derivative during local sensitivity computation. A technique which is commonly used to tune engineering systems is the root-locus method [34] which examines the trajectories of the roots or eigenvalues of a system when a particular parameter of interest is varied. The red curves in this figure incorporate the same information as a root-locus plot and the eigenvalue sensitivity represents the derivative of this relationship between eigenvalue and system parameter. The cyan line represents the nominal parameter setting ($k_{nominal}$).

The curves with blue markers in Fig. 8 are the eigenvalue sensitivity (${\hat{m}}$) plots. These curves cover the width of the bifurcation curve for each parameter. The ${\hat{m}}$ values in these plots represent the stability change in the system within the bistable region.

At steady state 1 ($SS_1$), $\lambda _{max}$ (red curve with triangular markers) is constant for perturbation in $k_1$, $k_2$, or $k_3$; therefore, the eigenvalue sensitivity (blue curve with triangular markers) is zero (Fig. 8a-c). At the same time, $\lambda _{max}$ variation w.r.t. $k_4$ is a linear function resulting in unity eigenvalue sensitivity (Fig. 8d). These global trends are correlated with the local sensitivity analysis presented earlier in Fig. 3a.

At steady state 2 ($SS_2$), $\lambda _{max}$ (red curve with square markers) is not a linear function of any of the parameters, so eigenvalue sensitivity ${\hat{m}}$ (blue curve with square markers) is not constant throughout the bistable region. In this case, the local sensitivity analysis at $k_{nominal}$ (Fig. 3a) is only a linear approximation of the non-linear global trend in the bistable region; however, the sign of ${\hat{m}}$ is accurate and reliable for making switching decisions (the design rules of Table 2 are consistent with the global trend). Eigenvalue sensitivity has positive values for $k_1$ and $k_2$, and is negative for $k_3$ and $k_4$.

### Scalability of eigenvalue sensitivity and separation sensitivity analyses

**Fig. 9:**
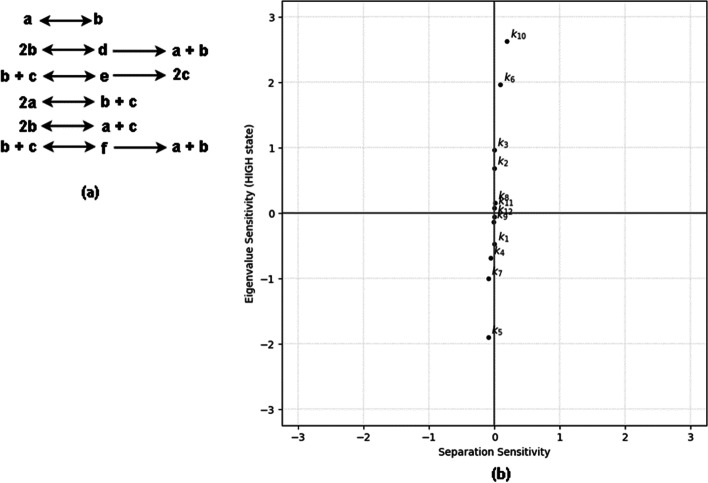
Demonstrating eigenvalue sensitivity and separation sensitivity analyses on a 12-parameter bistable system (a) Reactions for a larger bistable system from [42]. See Additional file 1 for the system of ODEs and parameter values for this system. (b) The plot shows eigenvalue sensitivity and separation sensitivity for the 12 parameters. Low values of separation sensitivity across all parameters indicate a good bistable switch

**Fig. 10:**
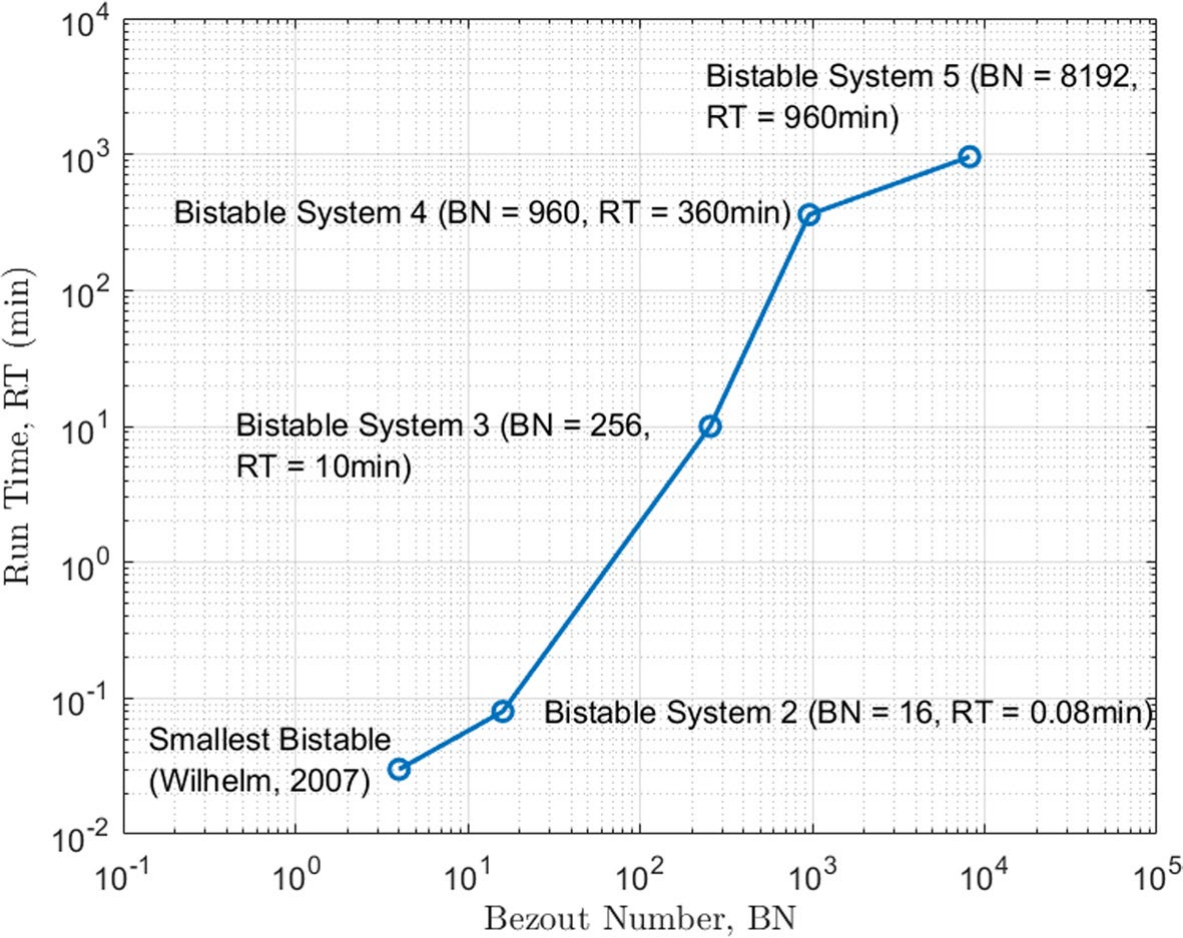
Bezout Number vs. Run time of different bistable system models where sensitivity analyses were implemented. The x-axis shows Bezout number [48] which is the number of solutions of the system computed as the product of the polynomial orders of the system (for example, if we were to determine the solution for the intersection of two circles, the Bezout number is 4 which is the product of maximum degrees of two quadratic equations). Note that both axes are in log scale. Y-axis is the run time in minutes to determine the solution of the model

We applied the proposed sensitivity analyses to a few larger (number of species greater than five and number of reactions greater than six) bistable systems from a library [42] of such systems with mass-action kinetics. An example of such a bistable system has 6 ODEs consisting of 12 parameters. Our sensitivity analyses results in Fig. 9 show that the bistable system has constant separation between stable states because the separation sensitivity across all parameters cluster close to zero, indicating that the switch is robust. Additionally, the eigenvalue sensitivity in that figure shows that the most sensitive parameters are $k_6$ and $k_{10}$ which are reaction constants belonging to a positive feedback loop consisting of autocatalysis and free-radical combination reactions. This application illustrates that our methodology can be applied to a larger class of bistable systems with ODEs using mass-action kinetics. In our software pipeline, the most computationally expensive steps to analyze these systems are homotopy continuation (used to compute steady states by solving a linear system of equations shown in line numbers 8 and 20 in Algorithm. 1) and eigenvalue analysis (shown in line numbers 16 and 28 in Algorithm. 1). We show in Fig. 10 how the computation time increases with respect to the size of the ODE system measured in terms of the Bezout number [48]. In this section we have shown a few illustrations of systems that can be analyzed using these sensitivity methods; a complete description of such systems is beyond the scope of this work.

## Discussion

Systems biologists have relied on classic approaches such as bifurcation and phase plane analysis to analyze bistable systems. While those methods convey qualitative information about a system, eigenvalue sensitivity and stable state separation sensitivity analyses that we presented in this work are scalable quantitative techniques to provide insights regarding a system’s parameter dependence.

Eigenvalue sensitivity is the rate at which parameter perturbation affects stability. Stable state separation sensitivity is the change in Euclidean distance between stable states as parameters are changed; it represents the sensitivity of noise immunity. Separation sensitivity looks at the whole system while eigenvalue sensitivity is specific to each stable state. The signs of these measures indicate stabilizing or destabilizing trends depending on the direction of parameter change. Both these measures are dimensionless and, therefore, do not require the system to be non-dimensionalized.

Sensitivity analyses can help in designing a bistable switch to make desirable state transitions as shown in Fig. 6. Furthermore, sensitivity analyses can expose stability behavior that may not be observable from the bifurcation plots. For example, it is not evident from the bifurcation plots (Fig. 1d) that perturbations in $k_1$, $k_2$, $k_3$ do not affect the stability of the OFF state (or the low steady state). Separation sensitivity by itself provides limited information; however, together with eigenvalue sensitivity, it helps in identifying dominant parameters that govern the stability of the bistable system as shown in Fig. 6. This allows to control the system by optimally ‘dialing in’ the dominant parameters. We demonstrated such control of a switch using sensitivity measures on the simplest published bistable system.

Our goal with this work was to propose eigenvalue sensitivity analysis and separation sensitivity analysis as a framework to evaluate large and complex bistable systems. Demonstration using the two-component tractable example validates the analyses because parameter design of a minimal bistable system can be done intuitively by manual inspection.

When parameters of such large-scale systems need to be modified to make desirable state transitions, it can be hard to visually inspect the parameter bifurcation diagrams and make qualitative decisions. In such cases, parameter clustering based on quantitative sensitivity analyses presented in this work can be valuable. Eigenvalue and separation sensitivity are signed measures and we showed that a clustering of parameters using the sign of these measures provides design rules for bistable systems.

Application of these analyses to large-scale and complex bistable systems is valuable but not without challenges. The principal computational modules for techniques discussed here are root-finding for algebraic systems (to compute steady-states), solution of eigenvalue problems, and gradient computation (to compute sensitivities). In this work, we have used homotopy continuation for root-finding [48] which has also been demonstrated for larger-scale algebraic systems. Solutions for steady states and the eigenvalue problem need to be determined three times for each parameter (one for the nominal setting and one each for the bidirectional perturbations), which can be computationally intensive. We recommend using parallel computing as discussed in [42]. Sensitivity analysis relies on finite difference methods that may introduce numerical noise in the gradient estimates; however optimal stepping algorithms [49] can be used to improve gradient accuracy for large-scale systems.

In models where the system has some components which are not reducible to a Jacobian (e.g., signaling models with algebraic relationships between species, see models from the DOCQS database [50]) more sophisticated tools such as homotopy continuation will be needed to map such systems to simpler systems for which we can determine a Jacobian and conduct the analysis as done here. This is beyond the scope of this paper.

In our analysis of the simplest bistable system, it became evident that it is important to consider the proximity of a nominal parameter setting to the edges of the bistable region in the bifurcation plots. We hypothesize that it is possible to measure this based on the eigenvalue sensitivity measure and nominal parameter set. For instance, using the first-order sensitivity and the maximum eigenvalue at the nominal parameter setting, we could iteratively estimate the parameter value at which bistability will be lost ($\lambda _{max} \approx 0$). This new measure could then tell us how close the nominal parameter setting is to the “cliff” of bistable bifurcation curve. Doing this on both sides of the parameter range, we can estimate the parameter range for bistability. This could also potentially be a measure for dialing in the optimal parameter set.

## Conclusion

Many mechanisms in biology are governed by underlying bistable systems. This warrants designing bistable systems to regulate or synthesize such mechanisms. In this work, we propose eigenvalue sensitivity analysis as a metric which can determine the most sensitive parameter of a bistable system. Eigenvalue sensitivity is computed as rate of change of eigenvalues with respect to change in parameter. The most sensitive parameter(s) can take the bistable system to a desirable state with minimal amount of perturbation. Similarly we introduce stable state separation sensitivity analysis to determine how far apart the stable states are. Both these measures together dictate how easy it is to regulate a bistable system.

We applied our analysis to the smallest bistable system [41] and found two parameters having more sensitivity in regulating stable states of this system. We demonstrated that these parameters are indeed most easily modified in comparison to other parameters, to switch from a bistable steady state to a monostable steady state. Many therapeutic researches such as Cancer Pharmacology consider alternatives to design bistable systems to retain or to lose bistability.

## Methods

### Dynamical system models for chemical reactions networks

The dynamics of a chemical reaction network are described using a set of reaction rate equations:

3 $$\begin{aligned} {\dot{\textbf{x}}} = f\left( \textbf{x},\textbf{p},t\right) \text {, } \textbf{x}(0) = \textbf{x}_0 \end{aligned}$$

where $\textbf{x}\in \mathbb {R}^{n+}$ represents the species concentrations and $\textbf{p}\in \mathbb {R}^{m+}$ is a system parameter vector which is usually constant for a given model. For a bistable network, there are three steady states $\textbf{x}_{ss}$ for the system in Eq. 3, of which two are stable and one is a saddle node [51]. To determine stability, the Jacobian matrix ($\textbf{A}\in \mathbb {R}^{n \times n}$) is computed by linearizing the system about each $\textbf{x}_{ss}$:

4 $$\begin{aligned} \Delta \dot{\textbf{x}}= \textbf{A}\Delta \textbf{x}\end{aligned}$$

When the eigenvalues of $\textbf{A}$ all have negative real parts, then the steady state is stable. If a system has at least two stable steady states, then the system is bistable and has the potential to behave like a biological switch. The maximum eigenvalue (spectral abscissa) represents how close the system is to instability. Such eigenvalue based classification is a basic step in understanding the stability of a system. We use homotopy continuation method using the HOMPACK package [48] to find steady state solutions of the system, and Maxima [52] to determine eigenvalues and stability of the corresponding steady state solutions. Subsequently, our algorithm generates the eigenvalue sensitivity vector, for each stable state, as described below.

### Algorithm for eigenvalue sensitivity analysis

The above analysis is carried out for two perturbations of a given parameter $p_j^* \in \textbf{p}$, one to each side of the nominal parameter value using $\epsilon _j$, a percentage perturbation scalar: $p_j^*(1-\epsilon _j)$ and $p_j^*(1+\epsilon _j)$. The eigenvalue sensitivity measure is computed, ${\hat{m}}_{ij}$, using the centered difference formula:

5 $$\begin{aligned} {\hat{m}}_{ij} = \left( \frac{\alpha ^{+}_{i} - \alpha ^{-}_{i}}{2 \epsilon _j p^{*}_j} \right) \frac{p^{*}_j}{\alpha ^{*}} \end{aligned}$$

where $\alpha ^+_{i}$ for the positive perturbation of the parameter about its nominal value is defined as:

6 $$\begin{aligned} \alpha ^+_{i} = \max \left[ \mathfrak {Re} \left( \pmb {\lambda }_i \right) \right] ^+ \end{aligned}$$

The vector $\pmb {\lambda }_i$ contains all the eigenvalues for steady state *i* and the mathematical operator $\mathfrak {Re}(z)$ denotes the real part of the complex number *z*. The maximum eigenvalue for the negative perturbation $\alpha ^-_{i}$ is defined similarly.

The procedure is repeated for all parameters of interest, one parameter at a time in a mutually exclusive manner (similar to one-parameter bifurcation analysis). The sensitivity values are clustered and ranked to determine the destabilizing parameter set and the dominant parameter within this set. The eigenvalue sensitivity measure ${\hat{m}}$ represents the polynomial order of the functional relationship between eigenvalue and given parameter, it is dimensionless, and signed. During post processing, the eigenvalue sensitivity vectors thus obtained can be used to determine (i) the cluster of parameters that de-stabilize the system and (ii) the most critical parameter that dictates the stability of the model.

The purpose of the computational method below is to generate a vector of eigenvalue sensitivities for each mathematical model when parameters are perturbed sequentially. Input into this algorithm (see below) is a bistable model with a specific set of parameter values called the nominal parameter set. The output is a vector of eigenvalue sensitivity with respect to each parameter of the system, for each stable state of the system. Eigenvalue sensitivity measures how much the model’s stability is influenced by perturbation in each of the selected parameters.

We measure the rate at which a particular parameter perturbation can “push” a bistable system into monostable region. Among all the parameters, we save the most sensitive parameter with the highest rate of change of eigenvalue (i.e. with maximum eigenvalue across both stable steady states). We compare eigenvalues corresponding to parameter changes in positive direction (i.e. right of nominal parameter value) with those in the negative direction (i.e. left of nominal parameter value). In each direction there are two stable steady states. So we ensure that we compared eigenvalues corresponding to the same stable steady states in either direction. This was to make sure that we are monitoring eigenvalues for systems that are in the vicinity of each other. We use Euclidean distance between steady states as a similarity measure to perform this check.
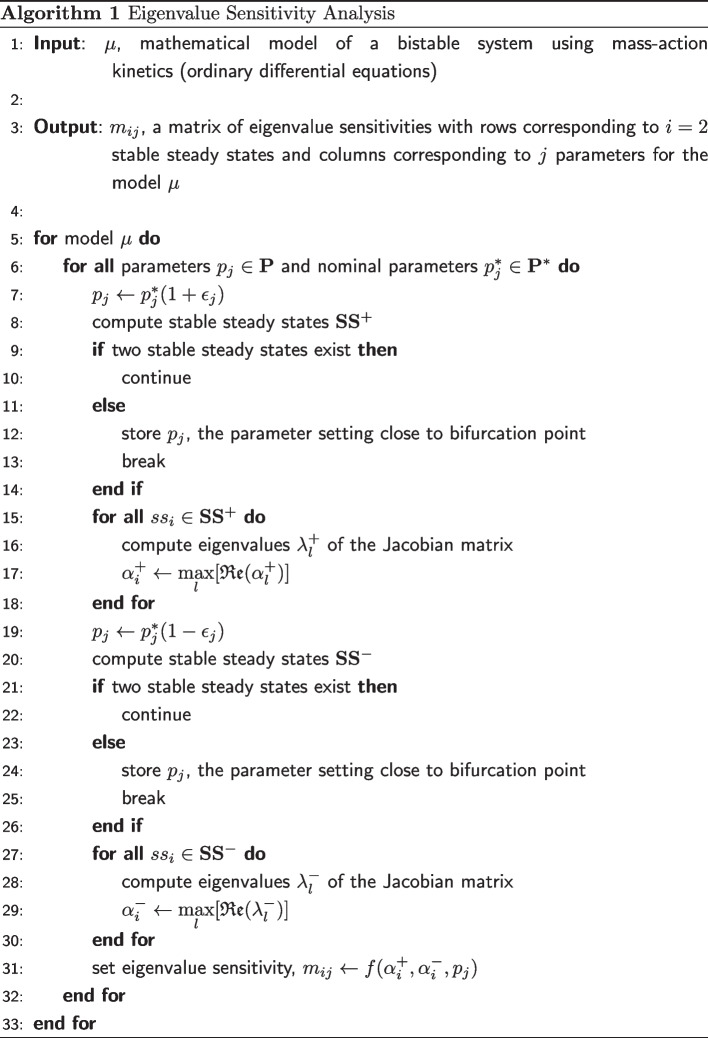


In Algorithm 1, the eigenvalue sensitivity metric can be described as a generic function:

7 $$\begin{aligned} m_{ij} = f(\alpha ^{+}_{i}, \alpha ^{-}_{i}, p_j) \end{aligned}$$

Equation 5 is a special case of Eq. 7 where the eigenvalue sensitivity is estimated using a centered difference method.

### Eigenvalue sensitivity measure

The eigenvalue sensitivity measure $m_{ij}$ between the *i*-th maximum eigenvalue ($\alpha _{i}$), and the *j*-th parameter ($p_j$), described in the algorithm section above, was analytically defined at a nominal operating point $(\alpha ^{*}_{i}, p^{*}_j)$ as:

8 $$\begin{aligned} m_{ij} = \frac{\partial {\alpha _i}}{\partial {p_j}} \frac{p^{*}_j}{\alpha ^{*}_i} \end{aligned}$$

Equation 8 may be re-written as:

9 $$\begin{aligned} m_{ij} = \left[ \frac{\partial {\ln (\alpha _i})}{\partial {\ln (p_j)}}\right] _{(\alpha ^{*}_i, p^{*}_j)} \end{aligned}$$

One advantage of formulating the sensitivity measure in this manner is that it is dimensionless. This allows us to directly compare eigenvalue sensitivities across multiple parameters, despite their fundamentally different units and orders of magnitude. One requirement for this measure is that the nominal maximum eigenvalue $\alpha ^{*}_i$ should be non-zero. This requirement is satisfied if we consider systems that are bistable to begin with. Such a measure for input–output sensitivity was proposed by Ferrell and Ha [39].

#### Proposition 1

If the nominal maximum eigenvalue $\alpha ^{*}_i$ is stable, then $m_{ij} > 0$ implies that a positive parameter perturbation $\Delta p_j$ stabilizes the maximum eigenvalue and a negative parameter perturbation $\Delta p_j$ destabilizes the maximum eigenvalue.

**Explanation**. Suppose we start our analysis with a stable nominal point (i.e. $\alpha ^{*}_i < 0$). Given that $p_j > 0 \text { } \forall j$, it follows from Eq. 8 that if $m_{ij} > 0$, then $sign(\frac{\partial {\alpha _i}}{\partial {p_j}}) = sign(\alpha ^{*}_i) = -1$.

Alternatively, the eigensensitivity $m_{ij}$ may be interpreted as a polynomial exponent based on Eq. 9:

10 $$\begin{aligned} \alpha _{i} = C (p_j)^{m_{ij}} \end{aligned}$$

where *C* is a constant, or,

11 $$\begin{aligned} \frac{\partial \alpha _{i}}{\partial p_j} = {m_{ij}} C (p_j)^{{m_{ij}}-1} \end{aligned}$$

This is equivalent to estimating the slope of the log-log plot. The slope indicates the polynomial exponent.

**Fig. 11:**
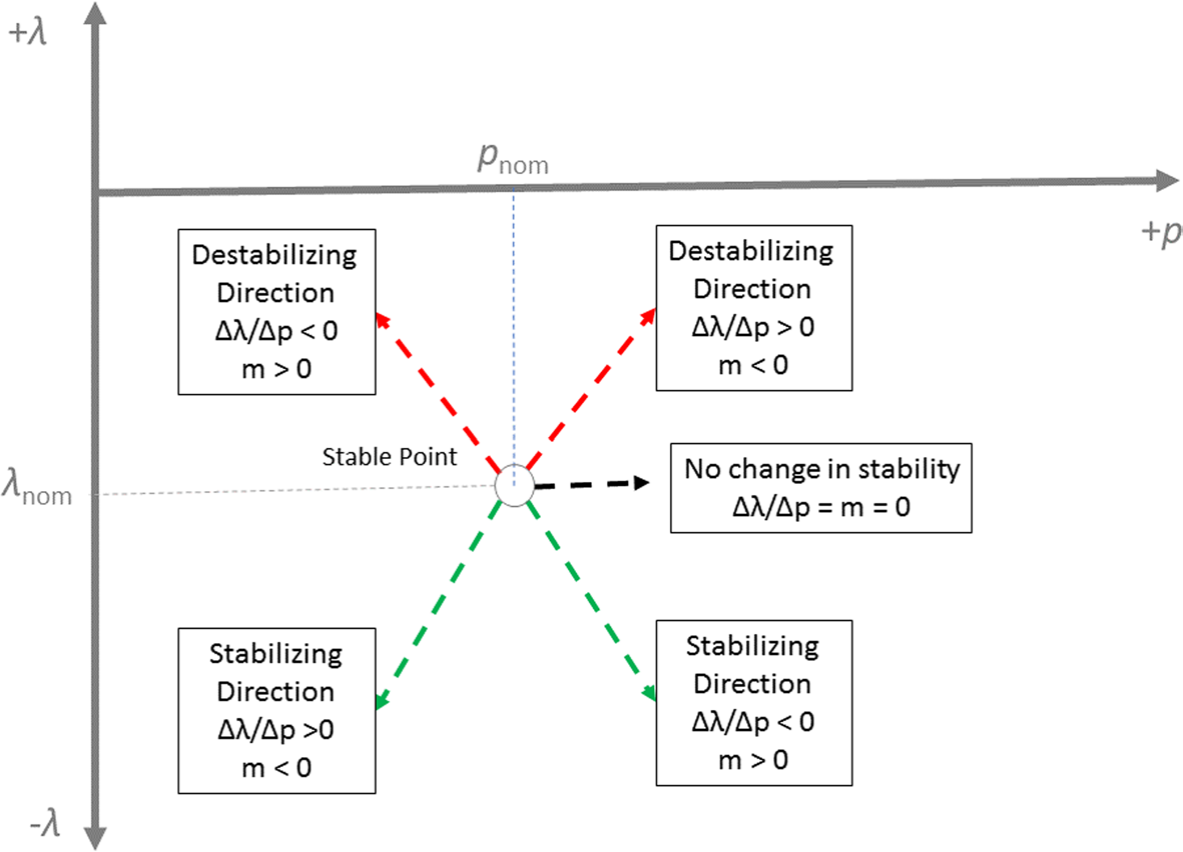
Interpretation of eigensensitivity measure *m* for a stable point. $m_{ij} > 0$ implies that the parameter should be increased ($\Delta p_j > 0$) to increase the stability of the system and it should be decreased to destabilize the system. Similarly, $m_{ij} < 0$ implies that the parameter should be decreased ($\Delta p_j < 0$) to increase the stability of the system and it should be increased to destabilize the system. The magnitude of *m* signifies how effective the parameter can be in stabilizing or destabilizing the system

In Eqs. 10–11, if $p_j > 0$, then $(p_j)^{m_{ij}} > 0$. Therefore, from Eq. 10, if our nominal point is stable, i.e. $\alpha ^{*}_i < 0$, then $C < 0$. From Eq. 11, we can therefore infer that when ${m_{ij}} < 0$, $\frac{\partial \alpha _{i}}{\partial p_j} > 0$, or destabilizing. Figure 11 illustrates the inverse relationship between these two quantities and enumerates all the possibilities to stabilize or destabilize a system using a parameter given the eigenvalue sensitivity.

### Finding output distribution of percent arc-length measure

In this section, we describe the domain of input and output for the Monte Carlo method used to determine the dominant parameter cluster for a population of models. The percent arc-length measure $s_r$ (introduced in the Results section) was described as:

12 $$\begin{aligned} s_r = s/s_{max} \end{aligned}$$

where *s* is the arc-length along the bifurcation curve in parameter-state space from a given stable state on the nominal model to the same stable state on the perturbed model (i.e. model with the perturbed parameter setting). The distance to cliff (or bifurcation point) from the nominal model is represented by $s_{max}$. The normalized distance to the cliff $s_c$ from the perturbed model is given by:

13 $$\begin{aligned} s_c = 1 - s_r \end{aligned}$$

From this formulation, it follows that for the nominal model $s_r=0$ (since $s=0$) and $s_c=1$. Similarly, at the bifurcation point $s_r=1$ because $s=s_{max}$, and $s_c=0$.

Consider a perturbation introduced in a given parameter $p_j+\Delta p_j$ for a population of models, all models beginning at the nominal parameter setting $p_j^{nom}$ in the ON state and perturbations for $p_j$ drawn from a log-normal distribution $log(p_j) \sim {\mathcal {N}}(\mu , \sigma ^2)$. If the maximum perturbation introduced in $p_j$ is $\Delta p_j^{max}$, then $\mu = p_j^{nom}$ and $3\sigma = \Delta p_j^{max}$. The minimum value (among the population of models) of normalized distance, $\min (s_c)$, to the cliff in the resulting output distribution is the distance of the maximally perturbed model to the bifurcation point. The above procedure is repeated for progressively increasing values of $\Delta p_j^{max}$ at equal steps and for all parameters. The parameter that achieved $\min (s_c) = 0$ with the lowest perturbation value $\Delta p_j^{max}$ was ranked the highest.

## Supplementary Information

**Additional file 1**. Bistable system models. This supplementary file includes mathematical as well as modeling details for the two bistable systems presented in this paper: (1) Chemical reaction network and system of ODE for the smallest bistable system (2) Eigenvalue analysis for the smallest bistable system (3) System of ODE for a larger bistable system.

## Data Availability

All data (system details, equations and algorithms) generated and/or analysed during this study are included in this published article.
