# Supplementary material for: Using sensitivity analyses to understand bistable system behavior
Source: BMC Bioinformatics. 2023 Apr 6;24:136. doi: 10.1186/s12859-023-05206-2 (PMC10080961; doi:10.1186/s12859-023-05206-2)
Supplement: Supplementary file 1 — Additional file 1. Bistable system models. This supplementary file includes mathematical as well as modeling details for the two bistable systems presented in this paper: (1) Chemical reaction network and system of ODE for the smallest bistable system (2) Eigenvalue analysis for the smallest bistable system (3) System of ODE for a larger bistable system. [file 12859_2023_5206_MOESM1_ESM.pdf]

Using sensitivity analyses to understand bistable system behavior

Vandana Sreedharan, Upinder S. Bhalla and Naren Ramakrishnan

Supplementary Information

Chemical reaction network

The chemical equations for smallest bistable system by Wilhelm [1] is shown below:

$$S + Y \xrightarrow{k_1} 2X \qquad \text{(Reaction 1)} \tag{1a}$$

$$2X \xrightarrow{k_2} X + Y \qquad \text{(Reaction 2)} \tag{1b}$$

$$X + Y \xrightarrow{k_3} Y + P \qquad \text{(Reaction 3)} \tag{1c}$$

$$X \xrightarrow{k_4} P \qquad \text{(Reaction 4)} \tag{1d}$$

The ODE system to model the dynamics of the above reactions is as follows:

$$\dot{x} = 2k_1 y - k_2 x^2 - k_3 xy - k_4 x \tag{2a}$$

$$\dot{y} = k_2 x^2 - k_1 y \tag{2b}$$

Species S and P are assumed to be constants in this model. S is incorporated in the value of k_1.

Eigenvalue Analysis

For the system in Eq. 2, there are three real and non-negative solutions (or steady states) from solving $\dot{x} = \dot{y} = 0$:

- $(x, y) = (0, 0)$
- $(x, y) = (\frac{-B \pm \sqrt{D}}{2A}, \frac{k_2 x^2}{k_1})$, where $B = -\frac{k_1}{k_3}$, $D = \left(\frac{k_1}{k_3}\right)^2 - 4\left(\frac{k_1 k_4}{k_3 k_2}\right)$, and $A = 1$

The system can be linearized about these steady states:

$$\dot{\mathbf{z}} = \mathbf{J}\mathbf{z} \tag{3}$$

where $\mathbf{z} = [\delta x, \delta y]^T$ and the Jacobian matrix is as follows:

$$\mathbf{J} = \begin{pmatrix} -2k_2 x - k_3 y - k_4 & 2k_1 - k_3 x \\ 2k_2 x & -k_1 \end{pmatrix} \tag{4}$$

The eigenvalues (λ_1, λ_2) of $\mathbf{J}$ can then be computed to determine if the steady state is stable, unstable, or a saddle node. Choosing the rate constants $k_1 = 8$, $k_2 = k_3 = 1$, and $k_4 = 1.5$ [1], the results of the above analysis are summarized in Table 1.

Table 1: Summary of eigenvalue analysis on the simplest bistable system [1]

	Solution 1	Solution 2	Solution 3
Steady States (x, y)	(0,0)	(2, 0.5)	(6, 4.5)
Jacobian $\mathbf{J}$	$\begin{pmatrix} -1.5 & 16 \\ 0 & -8 \end{pmatrix}$	$\begin{pmatrix} -6 & 14 \\ 4 & -8 \end{pmatrix}$	$\begin{pmatrix} -18 & 10 \\ 12 & -8 \end{pmatrix}$
Eigenvalues (λ_1, λ_2)	$(-1.5, -8)$	$(-14.5, 0.54)$	$(-25.04, -0.95)$
Stability Property	Stable	Saddle node	Stable

There are two stable steady states, which make this system bistable.

System of ODEs and parameter values for a larger bistable system from [2]

```
da/dt =  -1*k1*a + 1*k2*b + 1*k5*d -2*k9*a^2 + 2*ka*b*c + 1*kb*b^2 -1*kc*a*c + 1*kf*f
db/dt = 1*k1*a -1*k2*b -2*k3*b^2 + 2*k4*d + 1*k5*d -1*k6*b*c + 1*k7*e + 1*k9*a^2 -1*ka*b*c -2*kb*b^2 + 2*kc*a*c -1*kd*b*c + 1*ke*f + 1*kf*f
dd/dt = + 1*k3*b^2 -1*k4*d -1*k5*d
dc/dt =  -1*k6*b*c + 1*k7*e + 2*k8*e + 1*k9*a^2 -1*ka*b*c + 1*kb*b^2 -1*kc*a*c -1*kd*b*c + 1*ke*f
de/dt = + 1*k6*b*c -1*k7*e -1*k8*e
f =  (T0 - ( 1*a + 1*b + 1*c + 2*d + 2*e))/2

init a = 0.622176
init b = 0.985929
init c = 0.0160937
param T0 = 1.6242
param k1 = 0.163557
param k2 = 1.00155
param k3 = 93.5102
param k4 = 0.842684
param k5 = 0.210671
param k6 = 13.6847
param k7 = 13.5496
param k8 = 3.3874
param k9 = 0.307605
param ka = 0.160697
param kb = 1.11248
param kc = 1.29544
param kd = 0.46286
param ke = 1.16452
param kf = 0.29113
```

Figure 1: ODEs and parameter values for the bistable system in Figure 10. The reactions and parameter values are taken from [2]

Reference for Supplementary information

References

[1] Wilhelm, T.: The smallest chemical reaction system with bistability. BMC Systems Biology **3**(1), 90 (2009). doi:10.1186/1752-0509-3-90

[2] Ramakrishnan, N., Bhalla, U.S.: Memory switches in chemical reaction space. PLoS computational biology **4**(7), 1000122 (2008)
